# Molecular mechanism of c‐Myc and PRPS1/2 against thiopurine resistance in Burkitt's lymphoma

**DOI:** 10.1111/jcmm.15322

**Published:** 2020-05-11

**Authors:** Ting Li, Lili Song, Yingwen Zhang, Yali Han, Zhiyan Zhan, Zhou Xv, Yang Li, Yuejia Tang, Yi Yang, Siqi Wang, Shanshan Li, Liang Zheng, Yanxin Li, Yijin Gao

**Affiliations:** ^1^ Key Laboratory of Pediatric Hematology and Oncology Ministry of Health Department of Hematology & Oncology Shanghai Children’s Medical Center School of Medicine Shanghai Jiao Tong University Shanghai China

**Keywords:** Burkitt's lymphoma, c‐Myc, PRPS1/2, thiopurine resistance

## Abstract

Patients with relapsed/refractory Burkitt's lymphoma (BL) have a dismal prognosis. Current research efforts aim to increase cure rates by identifying high‐risk patients in need of more intensive or novel therapy. The 8q24 chromosomal translocation of the c‐Myc gene, a main molecular marker of BL, is related to the metabolism by regulating phosphoribosyl pyrophosphate synthetase 2 (PRPS2). In our study, BL showed significant resistance to thiopurines. PRPS2 homologous isoenzyme, PRPS1, was demonstrated to play the main role in thiopurine resistance. c‐Myc did not have direct effects on thiopurine resistance in BL for only driving PRPS2. PRPS1 wild type (WT) showed different resistance to 6‐mercaptopurine (6‐mp) in different metabolic cells because it could be inhibited by adenosine diphosphate or guanosine diphosphate negative feedback. PRPS1 A190T mutant could dramatically increase thiopurine resistance in BL. The interim analysis of the Treatment Regimen for Children or Adolescent with mature B cell non‐Hodgkin's lymphoma in China (CCCG‐B‐NHL‐2015 study) confirms the value of high‐dose methotrexate (MTX) and cytarabine (ARA‐C) in high‐risk paediatric patients with BL. However, there remains a subgroup of patients with lactate dehydrogenase higher than four times of the normal value (4N) for whom novel treatments are needed. Notably, we found that the combination of thiopurines and the phosphoribosylglycinamide formyltransferase (GART) inhibitor lometrexol could serve as a therapeutic strategy to overcome thiopurine resistance in BL.

## 1 INTRODUCTION

Burkitt's lymphoma (BL) is one of the highly invasive mature B cell malignant tumours, and its incidence rate is about 4.5/1 000 000. The genetic characteristic of BL is the 8q24 chromosomal translocation of the c‐Myc gene which is the main marker gene of BL.^1^ c‐Myc gene plays a crucial role in many important physiological activities such as cell division and proliferation, metabolism, cell cycle and apoptosis.^2^, ^3^ It has been found that the activated c‐Myc gene can induce the tumour metabolism. Therefore, the therapy of targeting c‐Myc is a trend in the treatment of high‐risk and relapsed or progressive BL.^4^

Davide Ruggero demonstrated that c‐Myc could regulate the metabolic changes of tumour cells by regulating the rate‐limiting enzyme phosphoribosyl pyrophosphate synthetase 2 (PRPS2).^5^ PRPS1 and PRPS2 which catalyse the same biochemical reaction ^6^ are critical rate‐limiting purine biosynthesis enzymes. However, PRPS1 has higher enzymatic and stronger allosteric activity than PRPS2.^7^ PRPS1 can also be significantly inhibited by adenosine diphosphate (ADP) and guanosine diphosphate (GDP) negative feedback.^8^ It was reported that negative feedback‐defective PRPS1 mutants could drive thiopurine resistance in relapsed childhood acute lymphoblastic leukaemia (ALL).^9^ It is unknown whether c‐Myc and PRPS2 are associated with the thiopurine resistance.

In the current study, we compared the characteristic of chemotherapy drug resistance in BL with that in leukaemia and dissected the roles of c‐Myc, PRPS1 and PRPS2 in thiopurine drug resistance in BL. BL showed great thiopurine resistance. PRPS1 played the key role in thiopurine resistance which showed different resistance to the thiopurines in different metabolic cells for the feedback inhibition of ADP/GDP. The interim analysis of the CCCG‐B‐NHL‐2015 study confirms the value of high‐dose (HD) MTX and ARA‐C in high‐risk paediatric patients with BL. However, there remains a subgroup of patients with lactate dehydrogenase (LDH) >4N for whom novel treatments are needed. The combination of thiopurine and the GART inhibitor lometrexol can increase the cell sensitivity to thiopurine resistance in BL. These results provide a new therapeutic strategy to overcome thiopurine drug resistance in BL.

## 2 MATERIALS AND METHODS

### 2.1 Cell culture

The Namalwa, Daudi, Raji BL cell lines and Reh, Molt4, Nalm6, Vocb6 leukaemia cell lines were cultured in RPMI 1640 medium supplemented with 10% foetal bovine serum (FBS) (HyClone), 100 U/mL penicillin G and 100 μg/mL streptomycin. HEK‐293T cells and human fibroblast (HF) cells were cultured in DMEM supplemented with 10% FBS (HyClone), 100 U/mL penicillin G and 100 μg/mL streptomycin. All cells were incubated at 37°C in 5% CO2 unless otherwise specified.

### 2.2 Cell construction

Phosphoribosyl pyrophosphate synthetase 1 WT, PRPS2 WT, c‐Myc coding DNA sequences were cloned into a pGV287 green fluorescent protein (GFP) vector. The PRPS1 A190T coding region was cloned into pGV303 GFP vector (Shanghai GeneChem). To produce the lentivirus, each expression vector was transfected into 293T cells with second‐generation lentiviral packaging plasmids pMD2. G and psPX2 using the PolyExpress transfection reagent (Excellgen). 48 and 72 hours after transfection, we harvested the culture medium, incubated with Lenti‐X concentrator (Clontech Laboratories), and centrifuged to obtain the concentrated lentivirus. The 293T cells were infected with the lentiviruses in the presence of 6 μg/mL polybrene (Sigma‐Aldrich) for 24 hours. Overexpression was confirmed by Western blot.

### 2.3 Western blot

Treated cells were harvested in lysis buffer and analysed by SDS‐PAGE with the following antibodies: c‐Myc (1:1000 dilution, Abcam, Cambridge, US), PRPS1 (1:1000 dilution, Santa, California, USA), PRPS2 (1:1000 dilution, Invitrogen, California, USA), flag‐tag (1:1000 dilution, Huaan, Hangzhou, China), his‐tag (1:1000 dilution, Huaan, Hangzhou, China) and actin (1:10 000 dilution, Huaan, Hangzhou, China). Immunoblots were analysed using the Odyssey system (LI‐COR Biosciences, Lincoln, Nebraska, USA).

### 2.4 Cell viability and apoptosis

Cell viability was determined by using the CellTiter‐Glo Luminescent kit (Promega, Madison, Wisconsin, USA) according to the manufacturer's instructions. Cells were seeded in 96‐well plates (10 000 cells per well) and treated for 72 hours with serially diluted drugs. CellTiter‐Glo reagents (50 μL) was added to each well and mixed for 10 minutes before the luminescence was measured on a microplate reader (BioTek, Vermont, USA). Apoptosis was measured by staining with annexin V‐APC and Propidium Iodide (PI)‐phycoerythrin (PE) (Annexin V‐APC Apoptosis Detection kit, BD Pharmingen), followed by flow cytometry on a FACS flow cytometer (BD, Canto II). All experiments were performed in triplicate, and results were calculated as the mean ± SD.

### 2.5 Stable gene knockdown

Lentiviral shRNAs were used to knock down c‐Myc expression in Raji, Daudi and Vocb6 cells. pLKO.1 shRNA vector and scrambled control pLKO.1 shRNA were purchased from Thermo Fisher Scientific. The knockdown efficiency of each shRNA was tested by Western blot. Clustered regularly interspaced short palindromic repeats (CRISPRs) were designed based on information available at http://crispr.mit.edu and were cloned into the lentiCRISPR/Cas9 vector (Addgene, Watertown, USA) by following the Zhang laboratory's protocol. The sequence targeted by PRPS1 CRISPR is 5′‐TTGGTCCTTACCAGGTCTCC‐3′, and the sequence targeted by PRPS2 CRISPR is 5′‐GGATGATGACGCAATCTTGC‐3′.

### 2.6 Metabolite flux

Cells were cultured for 48 hours in RPMI 1640, then harvested and pelleted. The reaction was quenched in cold 80% methanol, cells were centrifuged at 1200 g for 10 minutes, and metabolites in the supernatant were analysed by LC‐MS. The relative concentrations were defined according to the standard curve of compounds dissolved in 80% methanol without correcting for cell matrix effect.

### 2.7 Reverse transcription‐PCR analysis

MSH2 cDNA fragments containing the region between exon 9 and exon 16 were amplified by reverse transcription‐PCR with TaKaRa RNA PCR Kit (AMV) Ver.3.0 using Nalm6 and Reh total RNA as template. The primer sets were as follows:

Forward: 5’‐AAGCTGATTGGGTGTGGTCG‐3’.

Reverse: 5’‐TCGAAACCTCCTCACCTCCT‐3’.

### 2.8 Statistical analysis

All experiments were repeated three times. Data are presented as mean ± SD. Two‐tailed Student's *t* tests were performed, and *P* < 0.05 was considered statistically significant.

## 3 RESULTS

### 3.1 Drug resistance to thiopurines related to high c‐Myc expression as well as nucleotide metabolic level

**FIGURE 1:**
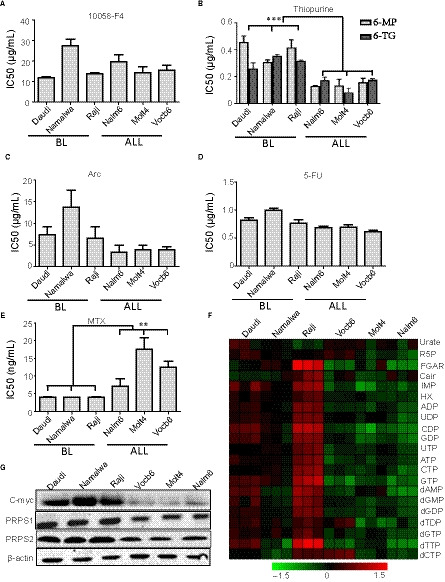
Drug resistance to thiopurines related to high c‐Myc expression as well as nucleotide metabolic level. A‐E, Viability of cells, including Burkitt's lymphoma cell lines (Daudi, Namalwa and Raji), ALL cell lines (Nalm6, Vocb6 and Molt4) at increasing concentrations of c‐Myc inhibitor 10058‐f4 (A), thiopurines (6‐mp/6‐TG) (B), ARA‐C (C), 5‐fu (D), MTX (E). F, Heatmap showing intracellular steady‐state metabolite profiles of Burkitt's lymphoma cell lines (Daudi, Namalwa and Raji), ALL cell lines (Nalm6, Vocb6 and Molt4). Red and green colours depict high and low nucleotide metabolism levels, respectively. Levels are scaled based on metabolism values that have been mean‐centred to zero. G, Representative Western blot analysis of c‐Myc and purine biosynthesis enzymes (PRPS1/2) in Burkitt's lymphoma cell lines (Daudi, Namalwa and Raji), ALL cell lines (Nalm6, Vocb6 and Molt4). β‐Actin was used as a loading control. ALL, acute lymphoblastic leukaemia

In BL, some patients do not respond to initial therapy or relapse after the standard therapy, which leads to poor prognosis. The mechanisms underlying BL chemoresistance remain poorly defined. To uncover the intrinsic mechanism, we used several cell lines of BL (Daudi, Namalwa and Raji) and ALL (Molt4, Vocb6 and Nalm6) as model. First, we detected half inhibit concentration (IC50) of several key chemotherapy drugs, such as 6‐mercaptopurine(6‐mp), 6‐thioguanine (6‐TG), cytarabine (ARA‐C), methotrexate (MTX),^10^ 5‐fluorouracil (5‐FU) and c‐Myc inhibitor 10058‐f4 in all cell lines (Figure 1A‐E). We found that there was no difference on drug resistance to 5‐FU, ARA‐C and 10058‐f4 in two disease cell lines and MTX showed more sensitive in BL than in ALL cell lines (Figure 1A,C,D,E). According to our results, the resistance to thiopurines, 6‐mp and 6‐TG, was significantly higher in BL cells than in ALL cell lines (Figure 1B). We then examined the level of nucleotide metabolism in all cell lines because thiopurines targeted at the purine metabolic pathway. BL cells displayed the increased levels of nucleotide metabolism including purine and pyrimidine nucleotides, especially ADP, GDP, inosine monophosphate (IMP), hypoxanthine (HX) and so on (Figure 1F).

The most important genetic characteristic of BL is the amplification of the c‐Myc gene which regulates PRPS2 expression to drive tumour metabolism.^1^, ^5^ We detected the protein expression of c‐Myc and PRPS1/2 in different cell lines. The protein expression of c‐Myc and PRPS1/2 in BL cells was significantly increased. (Figure 1G). Therefore, it suggested that the high expression of c‐Myc gene led to the high level of nucleotide metabolism resulting in the thiopurine resistance in BL.

### 3.2 Direct manipulation of c‐Myc has no effects on thiopurine drug resistance

**FIGURE 2:**
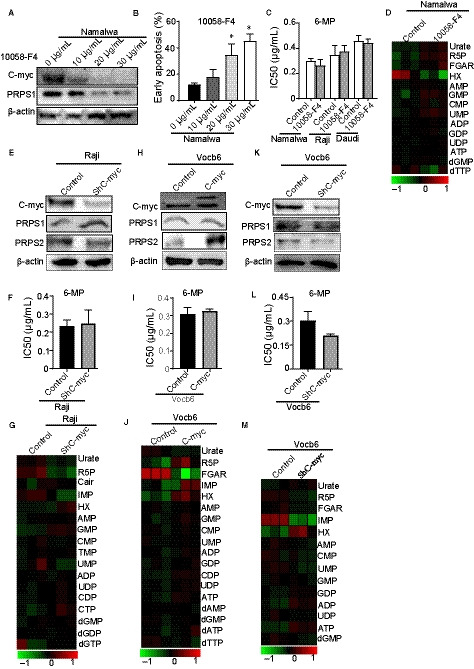
Direct manipulation of c‐Myc has no effects on thiopurine drug resistance. A, Western blot analysis of oncogene c‐Myc in Namalwa after treating with increasing concentrations of c‐Myc inhibitor 10058‐f4 (0, 10, 20, 30 µg/mL) for 48 h. β‐Actin was used as a loading control. B, Apoptosis of Namalwa after treating for 48 h with increasing concentrations of c‐Myc inhibitor 10058‐f4 (0, 10, 20, 30 µg/mL). *: *P* < 0.05; two‐tailed Student's *t* tests. C, The 6‐mpIC50 of Burkitt's lymphoma cells including Namalwa, Daudi and Raji treated with or without 20 µg/mL c‐Myc inhibitor 10058‐f4 for 48 h. *P* > 0.05; two‐tailed Student's *t* tests. D, Heatmap showing metabolomics analysis of Namalwa after treating with or without 20 µg/mL c‐Myc inhibitor 10058‐f4. E, c‐Myc and PRPS1/2 in a Western blot analysis of sh‐c‐Myc Raji cells. β‐Actin was used as a loading control. f, Cell viability of sh‐c‐Myc Raji cells at increasing concentrations of 6‐mp. *P* > 0.05; two‐tailed Student's *t* tests. G, Heatmap showing intracellular steady‐state metabolite profiles of sh‐c‐Myc Raji cells. H, Western blot of c‐Myc and PRPS1/2 in c‐Myc overexpressed Vocb6cells. β‐Actin was used as a loading control. I, The IC50 of thiopurines in c‐Myc overexpressed Vocb6cells. *P* > 0.05; two‐tailed Student's *t* tests. J, Heatmap showing intracellular steady‐state metabolite profiles of cell lines in (H). K, Western blot of c‐Myc and PRPS1/2 in sh‐c‐MycVocb6cells. β‐Actin was used as a loading control. L, Testing the 6‐mpIC50 of Vocb6sh‐c‐Myc cell line. *P* > 0.05; two‐tailed Student's *t* tests. M, Heatmap showing purine metabolite profiles of cell lines in (K)

To confirm that c‐Myc was related to thiopurine drug resistance, we first used three different concentrations of a small molecular c‐Myc inhibitor, 10058‐F4, to decrease the level of c‐Myc in BL by inhibiting the c‐Myc/Max heterodimerization (Figure 2A).^11^ And the high concentration of the c‐Myc inhibitor could dramatically increase cell apoptosis (Figure 2B). We then analysed the effects of different concentrations of 10058‐f4 in response to 6‐mp in different BL cell lines. According to our data, no matter what concentration of 10058‐f4 was, IC50 of 6‐MP showed no significant decrease (Figure S1A). What's more, the combination of 10058‐f4 and 6‐mp did not affect the thiopurine resistance in all BL cells (Figure 2C). Identical with the data of drug sensitivity, the nucleotide metabolic level in BL cells showed no difference between cells treated with and without 10058‐f4 (Figure 2D). It suggested that c‐Myc may have no direct function in 6‐mp drug resistance.

Then, we knocked down c‐Myc in high metabolic BL cell lines (Figure 2E, Figure S2A) and low metabolic Vocb6 ALL cell line (Figure 2K), whereas we overexpressed c‐Myc in low metabolic ALL cell lines (Figure 2H, Figure S2C). The c‐Myc knockdown had little effects on thiopurine drug resistance (Figure 2F,l, Figure S2B), and the c‐Myc overexpressed cell lines conferred thiopurine resistance similarly to control cells as well (Figure 2I, Figure S2D). Then, we detected the level of nucleotide metabolism in c‐Myc regulated BL and ALL cell lines mentioned above. The regulation of c‐Myc also had faint influences on purine metabolism (Figure 2G,j,m, Figure S2E). Therefore, our results suggested that direct regulation of c‐Myc did not affect thiopurine drug resistance. What's more, PRPS2 may have no function on thiopurine resistance, and some other molecules regulating the purine metabolism remain uncovered.

### 3.3 PRPS1 plays the key role in the thiopurine resistance regulated by the level of purine metabolites such as IMP, ADP and GDP

**FIGURE 3:**
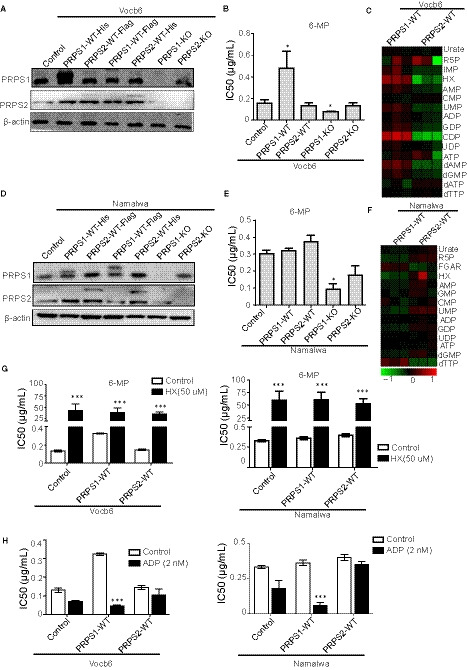
PRPS1 plays the key role in the thiopurine resistance regulated by the level of purine metabolites such as HX, IMP and ADP or GDP. A, Western blot of PRPS1/2 in Vocb6 cell lines including control, PRPS1‐WT‐His, PRPS2‐WT‐Flag, PRPS1‐WT‐Flag, PRPS2‐WT‐His, PRPS1 KO and PRPS2 KO. β‐Actin was used as a loading control. B, The sensitivity to 6‐mp in Vocb6 cell lines including control, PRPS1‐WT, PRPS2‐WT, PRPS1 KO and PRPS2 KO. *:*P* < 0.05; two‐tailed Student's *t* tests. C, Heatmap showing purine metabolic levels of PRPS1‐WT and PRPS2‐WTVocb6 cell lines. D, Testing the PRPS1/2 level in Namalwa cell lines including control, PRPS1‐WT‐His, PRPS2‐WT‐Flag, PRPS1‐WT‐Flag, PRPS2‐WT‐His, PRPS1 KO and PRPS2 KO via Western blot. β‐Actin was used as a loading control. E, Testing the 6‐mpIC50 of Namalwa cell lines including control, PRPS1‐WT, PRPS2‐WT, PRPS1 KO and PRPS2 KO. *:*P* < 0.05; two‐tailed Student's *t* tests. F, Heatmap showing purine metabolic levels of PRPS1‐WT and PRPS2‐WT Namalwa cell lines. G, Testing the sensitivity of 6‐mp in Namalwa and Vocb6 cell lines including control, PRPS1‐WT, PRPS2‐WT after treating with or without 50 µmol/L HX. *:*P* < 0.05; two‐tailed Student's *t* tests. H, Testing the sensitivity of 6‐mp in Namalwa and Vocb6 cell lines including control, PRPS1‐WT, PRPS2‐WT after treating with or without 2nMADP. *:*P* < 0.05; two‐tailed Student's *t* tests. ADP, adenosine diphosphate; GDP, guanosine diphosphate; HX, hypoxanthine; IMP, inosine monophosphate; PRPS1, phosphoribosyl pyrophosphate synthetase 1; PRPS2, phosphoribosyl pyrophosphate synthetase 2

On the basis of the role of PRPS1 and PRPS2 in purine biosynthesis, we examined whether the regulation of PRPS1 or PRPS2 had the same thiopurine drug resistance in different metabolic cell lines. We established ALL and BL cell lines infected with PRPS1 wild type (1‐wt), PRPS2 wild type (2‐wt), PRPS1 knocked out (1‐ko) and PRPS2 knocked out (2‐ko) retroviruses (Figure 3A,D, Figure S3A,C,E). In low purine metabolic ALL cell lines, the overexpression of PRPS1 obviously increased the level of thiopurine drug resistance, while overexpressed PRPS2 did not obviously change the 6‐mpIC50 (Figure 3B, Figure S3B). Moreover, knocking outPRPS1 in BL and ALL cell lines could dramatically decrease the 6‐mp drug resistance (Figure 3B,E, Figure S3B,D,F). We observed that Vocb6PRPS1‐WT cell line had higher purine metabolism than Vocb6PRPS2‐WT cell line as well (Figure 3C). As shown in Figure 3E, Figure S3D,F, overexpression of PRPS1 or PRPS2 in high metabolic cell lines showed little effects on thiopurine drug resistance. Moreover, similar to the ALL cell lines, knocking out PRPS1 in BL cell line could significantly decrease the IC50 of 6‐mp. Knockout of PRPS2 only slightly decreased the IC50 of 6‐mp (Figure 3E, Figure S3D,F). In addition, we investigated that PRPS2 WT showed no difference in purine metabolism with PRPS1 WT Namalwa cells, consistent with 6‐mp resistance data mentioned above (Figure 3F). The results suggested that PRPS1 played the main role in the thiopurine resistance, while PRPS2 did not affect the drug sensitivity significantly.

Nalm6 is a MSH2‐deficient precursor B cell lymphoblastic leukaemia (BCP‐ALL) cell line ^12^ (Figure S4A,B). MSH2‐deficient cell lines are highly sensitive to thiopurine drugs.^13^ Even though we overexpressed and knocked out PRPS1 and PRPS2 in the Nalm6 cell line (Figure S4C), the regulation of PRPS1 or PRPS2 slightly affected the thiopurine drug resistance (Figure S4D). Therefore, our data suggested that mutations which affected the stability of DNA could overcome the influence of regulating PRPS.

To understand why PRPS1 had different effects on thiopurine resistance in different metabolic BL and ALL cell lines, we examined the influence of nucleic acid metabolite ADP and HX on thiopurine resistance. First, we detected the sensitivity of HX and ADP in both BL and ALL cell lines to select appropriate concentrations (Figure S1B) and then tested the thiopurine resistance after adding HX or ADP. In all cancer cells, adding HX highly increased the resistance to thiopurine drugs in the same level (Figure 3G). Therefore, in BL, HX was significantly higher than that in ALL cell lines, which was one reason for drug resistance to 6‐mp (Figure 1F). Adding ADP significantly led to higher sensitivity of thiopurine drugs in all PRPS1 overexpressed cells, while PRPS2 overexpressing was resistant to the effects of ADP (Figure 3H). In BL, ADP and GDP were also significantly higher than those in ALL (Figure 1F). Therefore, overexpression of PRPS1 could not exhibit the enzymatic activity or affect the IC50 of 6‐mp. Our results suggested that high HX and ADP/GDP manipulated PRPS1 activity and the 6‐mp IC50. As a result, it implicates that PRPS1, not PRPS2, is the key factor in thiopurine resistance.

### 3.4 PRPS1 A190T is a resistant mutation of thiopurine by avoiding ADP or GDP inhibition

**FIGURE 4:**
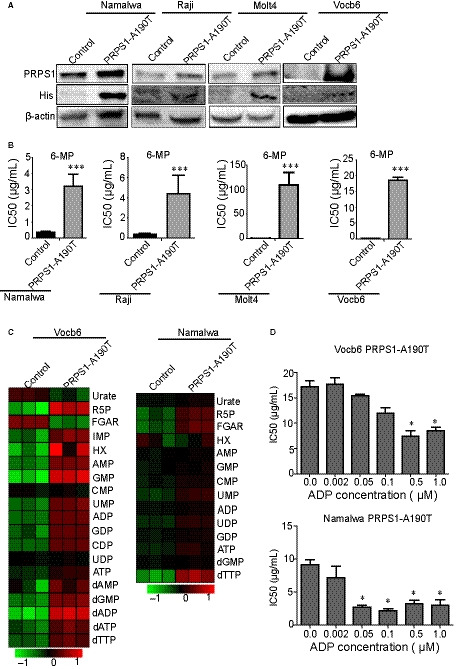
PRPS1 A190T is a resistant mutation of thiopurine by avoiding ADP or GDP inhibition. A, Western blot of the PRPS1 A190T mutant in Burkitt's lymphoma (Namalwa, Raji) and ALL cell lines (Molt4, Vocb6). β‐Actin was used as a loading control. B, The 6‐mpIC50 of PRPS1 A190T mutant Namalwa, Raji, Molt4 and Vocb6. Unregulated cells as control. *:*P* < 0.05; two‐tailed Student's *t* tests. C, Heatmap showing nucleotide metabolism of PRPS1 A190T mutant in Namalwa and Vocb6. D, The 6‐mp IC50 of PRPS1 A190T mutant Vocb6 and Namalwa at increasing concentrations of ADP (µmol/L).*: *P* < 0.05; two‐tailed Student's *t* tests. ADP, adenosine diphosphate; ALL, acute lymphoblastic leukaemia; GDP, guanosine diphosphate; PRPS1, phosphoribosyl pyrophosphate synthetase 1

Activating PRPS1 A190T mutant can escape nucleotide feedback inhibition, so PRPS1 A190T mutant can be a resistant mutation of 6‐mp in relapsed childhood ALL.^9^ To know whether the PRPS1 A190T mutant unlike PRPS1 WT showed the same drug resistance in BL as in ALL, we established the PRPS1 A190T mutation in BL and ALL cell lines (Figure 4A). We found that the PRPS1 A190T mutant presented significantly 6‐mp drug resistance in cell lines with different metabolic levels (Figure 4B). In agreement with this, the purine metabolic level of A190T mutant cells was much higher (Figure 4C). Moreover, the relative thiopurine resistance in high metabolic cell lines was much lower than that in low metabolic cell lines (Figure 4B). Then, we tested the different concentrations on the 6‐mp IC50 of PRPS1‐A190T mutant cells, and we found that the 6‐mp IC50 of PRPS1‐A190T mutant cells decreased at increasing concentrations of ADP (Figure 4D). PRPS1‐A190T in low metabolic cell lines could escape higher concentration of ADP than PRPS1‐A190T in high metabolic cell lines (Figure 4D). These data suggest that the concentration of ADP/GDP is important to the thiopurine resistance. When the concentration is high enough, ADP/GDP can also inhibit the function of PRPS1‐A190T. In a word, the thiopurine resistance of A190T mutations is limited.

### 3.5 The combination of thiopurines and lometrexol is benefit to BL treatment

**FIGURE 5:**
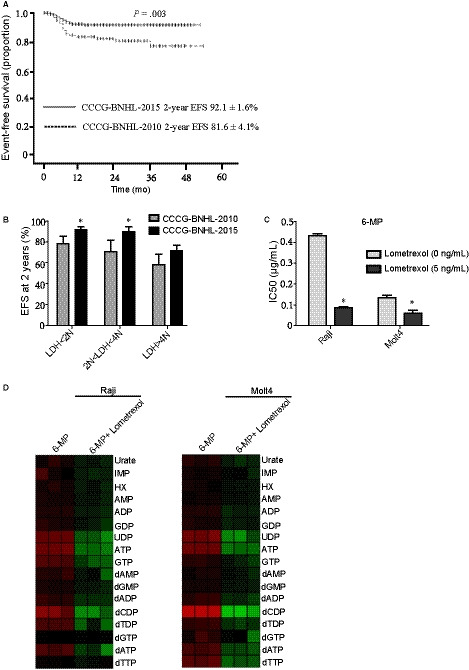
The combination of thiopurines and lometrexol is benefit to Burkitt's lymphoma treatment. A, EFS at 2 y for mature B cell non‐Hodgkin lymphoma paediatric patients in the two consecutive studies CCCG‐BNHL‐2015 (n = 314, interim analysis, unpublished data) and CCCG‐BNHL‐2010 (n = 104). *P* = 0.003. b, The 2‐year EFS of patients with different levels of LDH.N: the normal value. *P < 0.05; two‐tailed Student's *t* tests. C, The cell sensitivity to 6‐mp after treating with the 5 ng/mL GART inhibitor lometrexol. *P < 0.05; two‐tailed Student's *t* tests. D, Heatmap showing nucleotide metabolism after treating with 10 µg/mL 6‐mp or the combination of 10 µg/mL 6‐mp and 10 ng/mL lometrexol in Namalwa and Vocb6. EFS, event‐free survival; LDH, lactate dehydrogenase

It has been reported that HD MTX and Ara‐C are the major drugs in BL.^14^ Furthermore, our unpublished clinical trials put forward a new B‐NHL treatment programme called the CCCG‐B‐NHL‐2015 programme with HD MTX and ARA‐C (Tables 1 and 2). In the mid‐term summary, the B‐NHL‐2015 programme had a 2‐year event‐free survival rate (EFS) of 89.7 ± 1.8% compared to 76.1 ± 4.3% of the CCCG‐BNHL‐2010 programme (Figure 5A). Then, we used the LDH level of the B‐NHL patients to indicate the dose of c‐Myc expression. When the LDH level of the B‐NHL patients was less than 4 times of the normal value (4N), increasing the dose of MTX and Ara‐c could obviously prolong the 2‐year EFS. However, when LDH was higher than 4N, the B‐NHL‐2015 programme showed no significant difference from the B‐NHL‐2010 programme (Figure 5B). All these clinical data suggest that the therapy research targeting for its metabolic pathway is clinically reasonable and applicable.

**TABLE 1:** The CCCG‐B‐NHL‐2015 and CCCGB‐NHL 2010 programmes

Regimen	Risk group	Courses	Cumulative doses								
			CTX g/m^2^	IFOS g/m^2^	DOX mg/m^2^	MTX g/m^2^	ARA‐C g/m^2^	VP‐16 mg/m^2^	PRED/DEX mg/m^2^	VCR/VDS mg/m^2^	i.t.
CCCGB‐NHL 2010	G3	P+6	4.5	18	120	9	7	540	2835/—	18/—	13
	G4	P+6	3.1	12	140	6	4	960	1995/—	12/—	13
CCCG‐B‐NHL 2015	R3	P+6	4.5	18	120	15	12	900	2835/—	3/15	13
	R4	P+6	Based on regimen for R3, from first protocol BB, apply Rituximab, totally 4 times Rituximab (375 mg/m^2^)								13/15

**TABLE 2:**
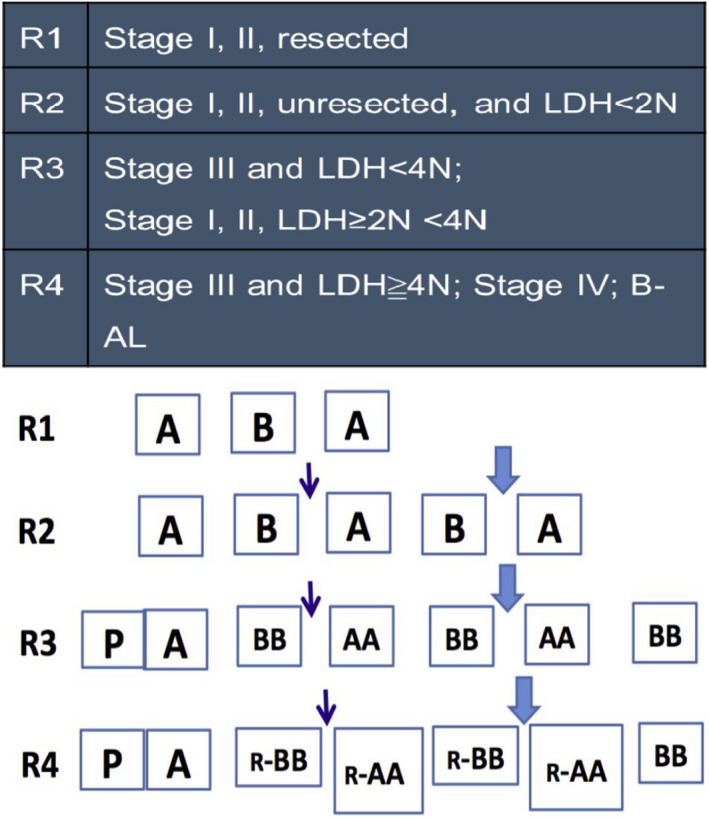
The stage and treatment protocol in the CCCG‐B‐NHL‐2015

Lometrexol (also called DDATHF) which is one of the GART inhibitors has been tested as an antitumour drug.^15^ We firstly detected the IC50 of lometrexol in BL and ALL cells to choose the appropriate concentration (Fig s1B) and then did combination studies. According to our results, the lometrexol treatment dramatically decreased the thiopurine resistance in BL cells to ALL cells level (Figure 5C). Next, we tested the nucleotide metabolic level after adding the combination of 6‐mp and lometrexol in BL and ALL cells. Compared to adding thiopurine only, the combination of thiopurine and lometrexol showed obviously lower metabolic levels in BL and ALL cells (Figure 5D). What's more, we observed that the lometrexol treatment could also decrease the thiopurine resistance in PRPS1 A190T mutant cells (Figure S1C). The IC50 of 6‐mp in normal human cells like HF cells was much higher than the concentration we used in cancer cells (Figure S1D). Simultaneously, the IC50 of the combination treatment in HF cells was much higher than that in cancer cells (Figure S1D). Therefore, this combination did not have any added toxic effects on normal cells. These data suggested that the combination of 6‐mp and lometrexol might serve as a therapeutic strategy to overcome thiopurine drug resistance in BL.

## 4 DISCUSSION

Patients with relapsed/refractory BL have a dismal prognosis. Current research efforts aim to increase cure rates by identifying high‐risk patients in need of more intensive or novel therapy. c‐Myc gene which is typically overexpressed in BL can regulate the metabolic changes of tumour cells by driving PRPS2. Our present study is to gain a better understanding of the roles of c‐Myc and PRPS1/2 in thiopurine drug resistance in BL to identify a more suitable molecular target and potentially improve the treatment of BL. Eventually, we found that the combination of thiopurines and the GART inhibitor lometrexol could overcome thiopurine drug resistance in BL which offered a new therapeutic strategy for the treatment of BL.

c‐Myc which encodes an important transcription factor affects many physiological processes such as cell proliferation, growth and metabolism by activating multiple target genes.^16^ The Mannava's laboratory found that knocking out the c‐Myc gene in human melanoma cell lines could reduce the expression of several rate‐limiting enzymes during nucleotide synthesis, such as thymidylate synthase, HX nucleotide dehydrogenase 2 (IMPDH2) and PRPS2 resulting in a decrease in intracellular nucleotide levels and cell proliferation.^17^ Moreover, in c‐Myc overexpressed cancer cells, the activated eukaryotic initiation factor 4E could bind with the pyrimidine‐rich translational element of PRPS2 mRNA to promote the translation of PRPS2 making the level of nucleotide metabolism up‐regulated.^5^ However, when we directly regulated the level of c‐Myc, the level of nucleotide metabolism was unchanged while the regulation had no effects on the resistance to thiopurine drugs. Considering the results we have achieved, it seems that c‐Myc cannot significantly regulate the nucleotide metabolism so it does not have direct relationship with the resistance to thiopurine drugs.

Phosphoribosyl pyrophosphate synthetase 1 and PRPS2 are homologous isoenzymes that catalyse the same biochemical reaction in both nucleotide de novo synthesis and salvage synthesis pathway.^18^ In 1995, Tatibana et al found that the recombinant expression of PRPS1 protein in vitro existed in the form of stable hexamers which could be significantly inhibited by ADP and GDP.^19^ Moreover, PRPS2 has lower enzymatic and allosteric activity than PRPS1, but is sensitive to cytokine regulatory responses.^20^ Consistently, our study showed that in BL cells, the function of PRPS1 was greatly inhibited by the high produced nucleotide metabolite like ADP and GDP. Overexpression of PRPS1 showed no influences on resistance to 6‐mp. On the contrary, in ALL cells with low metabolic level, PRPS1 with low ADP, GDP inhibition performed the key role in the thiopurine resistance. We also noticed that in either high or low metabolic cells, PRPS2 had almost no function on the thiopurine resistance for its low enzymatic and allosteric activity. And more, the regulation of PRPS1/2 in Nalm6 cell line did not obviously affect the sensitivity to thiopurine drugs. These data suggest that mutations affecting DNA stability can mask the effects of PRPS on drug resistance.

Phosphoribosyl pyrophosphate synthetase 1 A190T mutant which is a negative feedback‐defective PRPS1 mutant can be a resistant mutation of 6‐mpin relapsed childhood ALL.^8^ Although the A190T mutant showed significantly 6‐mp resistance in BL, the IC50 to thiopurine resistance was much lower than that in ALL cells. These data suggest that the PRPS1 A190T mutant can be inhibited if the level of nucleotide metabolism is high enough.

The French‐American‐British (FAB) Lymphomes Malins B studies demonstrated that HD MTX was an effective drug for CNS prophylaxis in BL.^21^, ^22^, ^23^, ^24^ The obligational Berlin‐Frankfurt‐Munster (BFM) studies also showed that the LDH level as c‐Myc marker was an important factor in the treatment of BL. The ongoing CCCG‐B‐NHL‐2015 confirmed those conclusions by increasing the dose of anti‐metabolic chemotherapeutic drugs. However, there remains a subgroup of patients with LDH >4N for whom novel treatment approaches are needed. In our study, the GART inhibitor lometrexol can increase the sensitivity of BL cells to thiopurines by inhibiting the nucleotide metabolism.

In summary, here we have reported a distinct role of c‐Myc and PRPS1/2 in thiopurine drug resistance in BL. Combination of thiopurines and the GART inhibitor lometrexol can overcome thiopurine drug resistance in BL. Therefore, our results not only clarify the role of c‐Myc and PRPS1/2 in the treatment of BL, but also offer a new therapeutic strategy for the treatment of BL.

## CONFLICT OF INTEREST

The authors declare that they have no conflicts of interest with the contents of this article.

## AUTHOR CONTRIBUTIONS

This study was conceived by YJ G and YX L.; YJ G and YX L. designed the study; T. L., LL S., YL, ZY Z, YW Z, YY, SQ W and SS L performed the experiments; T. L. analysed and interpreted the data; YJ G reviewed the manuscript; and TL and YX L. wrote the paper with comments from all authors. All authors read and approved the final manuscript.

## Supporting information

Fig S1

Fig S2

Fig S3

Fig S4

## Data Availability

All data supporting the findings of this study are available from the corresponding author on request.

## References

[1] Dalla‐Favera R , Bregni M , Erikson J , Patterson D , Gallo RC , Croce CM . Human c‐Myc onc gene is located on the region of chromosome 8 that is translocated in Burkitt lymphoma cells. Proc Natl Acad Sci USA. 1982;79(24):7824‐7827. 6961453 10.1073/pnas.79.24.7824 PMC347441

[2] Smith SM , Anastasi J , Cohen KS , Godley LA . The impact of MYC expression in lymphoma biology:beyond Burkitt lymphoma. Blood Cells Mol Dis. 2010;45(4):317‐323. 20817505 10.1016/j.bcmd.2010.08.002

[3] Satoh K , Yachida S , Sugimoto M , et al. Global metabolic reprogramming of colorectal cancer occurs at adenoma stage and is induced by MYC. Proc Natl Acad Sci USA. 2017;114(37):E7697‐E7706. 28847964 10.1073/pnas.1710366114 PMC5604037

[4] Lee S , Day NS , Miles RR , et al. Comparative genomic expression signatures of signal transduction pathways and targets in paediatric Burkitt lymphoma: a Children’s Oncology Group report. Br J Haematol. 2017;177(4):601‐611. 28474336 10.1111/bjh.14604 PMC5435544

[5] Cunningham JT , Moreno MV , Lodi A , Ronen SM , Ruggero D . Protein and nucleotide biosynthesis are coupled by a single rate‐limiting enzyme, PRPS2, to drive cancer. Cell. 2014;157(5):1088‐1103. 24855946 10.1016/j.cell.2014.03.052 PMC4140650

[6] Qian X , Li X , Tan L , et al. Conversion of PRPS hexamer to monomer by AMPK‐mediated phosphorylation inhibits nucleotide synthesis in response to energy stress. Cancer Discovery. 2018;8(1):94‐107. 29074724 10.1158/2159-8290.CD-17-0712 PMC5760453

[7] Becker MA , Heidler SA , Bell GI , et al. Cloning of cDNAs for human phosphoribosylpyrophosphate synthetases 1 and 2 and X chromosome localization of PRPS1 and PRPS2 genes. Genomics. 1990;8(3):555‐561. 1962753 10.1016/0888-7543(90)90043-t

[8] Chen P , Liu Z , Wang X , et al. Crystal and EM structures of human phosphoribosyl pyrophosphate synthase I (PRS1) provide novel insights into the disease‐associated mutations. PLoS ONE. 2015;10(3):e0120304. 25781187 10.1371/journal.pone.0120304 PMC4363470

[9] Li B , Li H , Bai Y , et al. Negative feedback‐defective PRPS1 mutants drive thiopurine resistance in relapsed childhood ALL. Nat Med. 2015;21(6):563‐571. 25962120 10.1038/nm.3840 PMC4670083

[10] Reiter A . Non‐Hodgkin lymphoma in children and adolescents. Klin Padiatr. 2013;225(S01):87‐93. 10.1055/s-0033-1337969 23700066

[11] Huang MJ , Cheng YC , Liu CR , Lin S , Liu HE . A small‐molecule c‐Myc inhibitor, 10058–F4, induces cell‐cycle arrest, apoptosis, and myeloid differentiation of human acute myeloid leukemia. Exp Hematol. 2006;34(11):1480‐1489. 17046567 10.1016/j.exphem.2006.06.019

[12] Suzuki T , Ukai A , Honma M , Adachi N , Nohmi T . Restoration of mismatch repair functions in human cell line Nalm‐6, which has high efficiency for gene targeting. PLoS ONE. 2013;8(4):e61189. 23596518 10.1371/journal.pone.0061189 PMC3626652

[13] Kynetski EY , Krynetskaia NF , Bianchi ME , Evans WE . A nuclear protein complex containing high mobility group proteins B1 and B2, heat shock cognate protein 70, ERp60 and glyceraldehyde‐3‐phosphate dehydrogenase is involved in the cytotoxic response to DNA modified by incorporation of anticancer nucleoside analogues. Cancer Res. 2003;63(1):100‐106. 12517784

[14] Minard‐Colin V , Brugières L , Reiter A , et al. Non‐Hodgkin lymphoma in children and adolescents: progress through effective collaboration, current knowledge, and challenges ahead. J Clin Oncol. 2015;33(27):2963‐2974. 26304908 10.1200/JCO.2014.59.5827 PMC4979194

[15] Adams J , Elliott PJ . New agents in cancer clinical trials. Oncogene. 2000;19(56):6687‐6692. 11426656 10.1038/sj.onc.1204088

[16] Liu YC , Li F , Handler J , et al. Global regulation of nucleotide biosynthetic genes by c‐Myc. PLoS ONE. 2008;3(7):e2722. 18628958 10.1371/journal.pone.0002722 PMC2444028

[17] Mannava S , Grachtchouk V , Wheeler LJ , et al. Direct role of nucleotide metabolism in C‐Myc‐dependent proliferation of melanoma cells. Cell Cycle. 2008;7(15):2392‐2400. 18677108 10.4161/cc.6390 PMC3744895

[18] An S , Kumar R , Sheets ED , Benkovic SJ . Reversible compartmentalization of de novo purine biosynthetic complexes in living cells. Science. 2008;320(5872):103‐106. 18388293 10.1126/science.1152241

[19] Tatibana M , Kita K , Taira M , et al. Mammalian phosphoribosyl‐pyrophosphate synthetase. Adv Enzyme Regul. 1995;35(1):229‐249. 7572345 10.1016/0065-2571(94)00017-w

[20] Becker MA , Smith PR , Taylor W , Mustafi R , Switzer RL . The genetic and functional basis of purine nucleotide feedback‐resistant phosphoribosylpyrophosphate synthetase superactivity. J Clin Invest. 1995;96(5):2133‐2141. 7593598 10.1172/JCI118267 PMC185862

[21] Holt SM , Scemama J‐L , Panayiotidis MI , Georgakilas AG . Compromised repair of clustered DNA damage in the human acute lymphoblastic leukemia MSH2‐deficient NALM‐6 cells. Mutat Res. 2009;674(1‐2):123‐130. 18955159 10.1016/j.mrgentox.2008.09.014

[22] Patte C , Philip T , Rodary C , et al. High survival rate in advanced‐stage B‐cell lymphomas and leukemias without CNS involvement with a short intensive polychemotherapy: results from the French Pediatric Oncology Society of a randomized trial of 216 children. J Clin Oncol. 1991;9(1):123‐132. 1985161 10.1200/JCO.1991.9.1.123

[23] Patte C , Auperin A , Michon J , et al. The Société Française d’Oncologie Pédiatrique LMB89 protocol: highly effective multiagent chemotherapy tailored to the tumor burden and initial response in 561 unselected children with B‐cell lymphomas and L3 leukemia. Blood. 2001;97(11):3370‐3379. 11369626 10.1182/blood.v97.11.3370

[24] Woessmann W , Seidemann K , Mann G , et al. The impact of the methotrexate administration schedule and dose in the treatment of children and adolescents with B‐cell neoplasms: a report of the BFM Group Study NHL‐BFM95. Blood. 2005;105(3):948‐958. 15486066 10.1182/blood-2004-03-0973

