# Supplementary figures and images for: Molecular mechanism of c‐Myc and PRPS1/2 against thiopurine resistance in Burkitt's lymphoma
Source: J Cell Mol Med. 2020 May 11;24(12):6704–15. doi: 10.1111/jcmm.15322 (PMC7299692; doi:10.1111/jcmm.15322)

Fig.S1

a

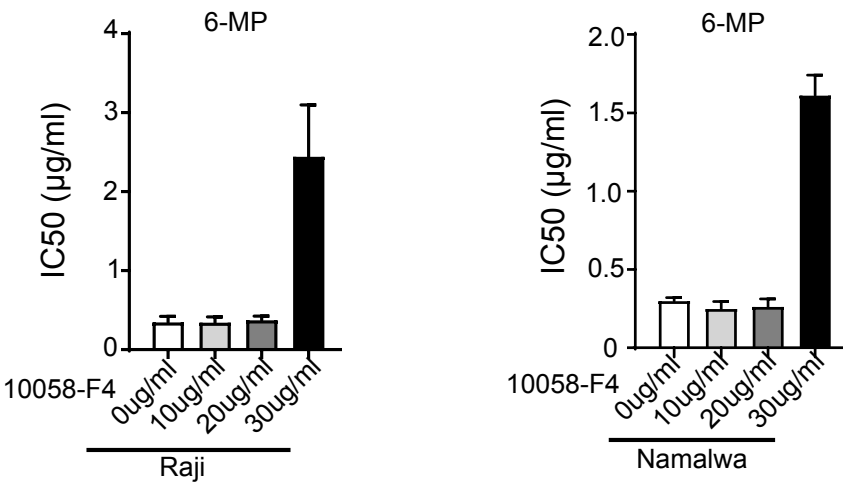

b

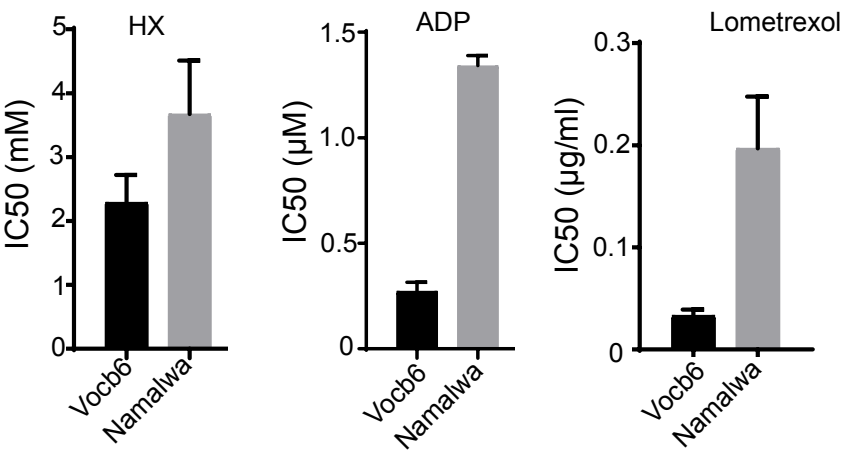

c

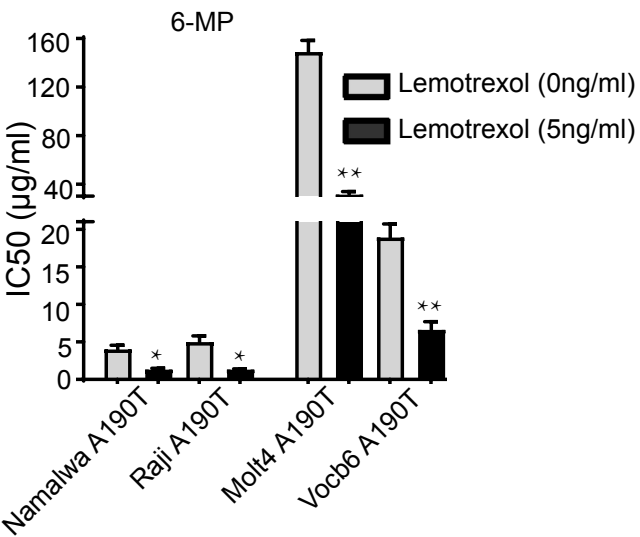

d

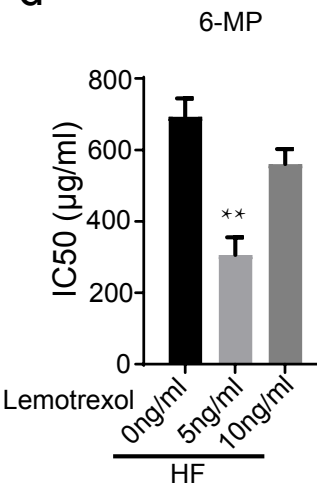

Supplement: Supplementary file 1 — Fig S1 [file JCMM-24-6704-s001.pdf]

Fig.S2

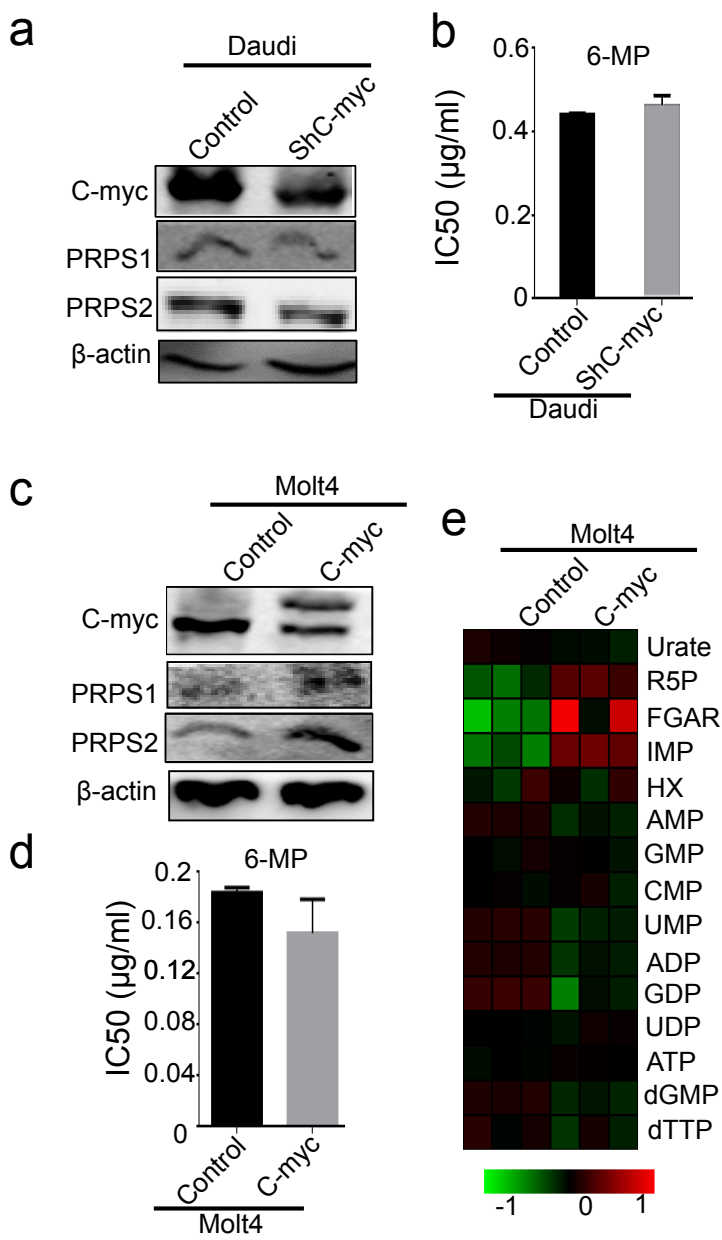

Supplement: Supplementary file 2 — Fig S2 [file JCMM-24-6704-s002.pdf]

Fig.S3

a

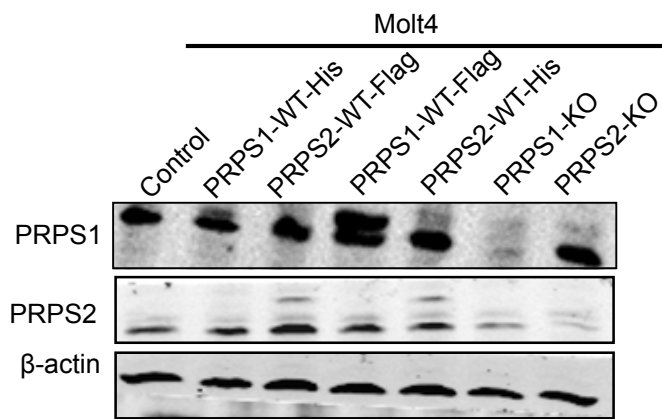

b

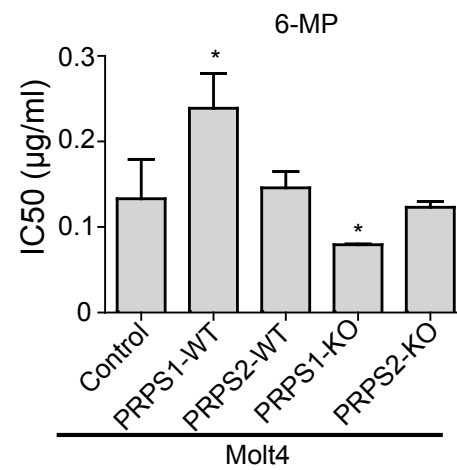

c

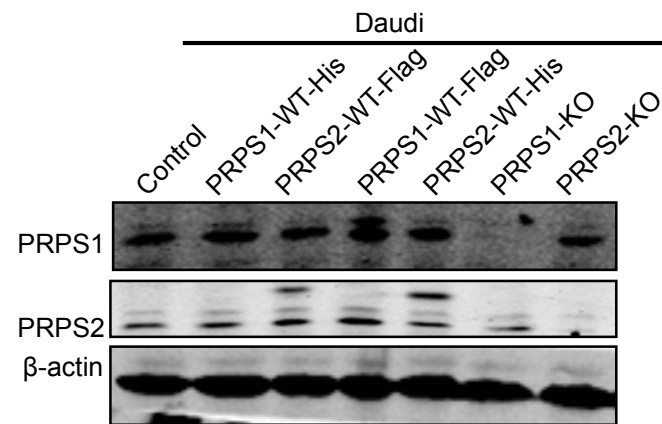

d

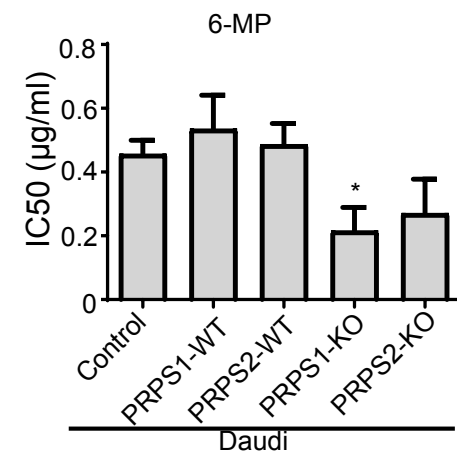

e

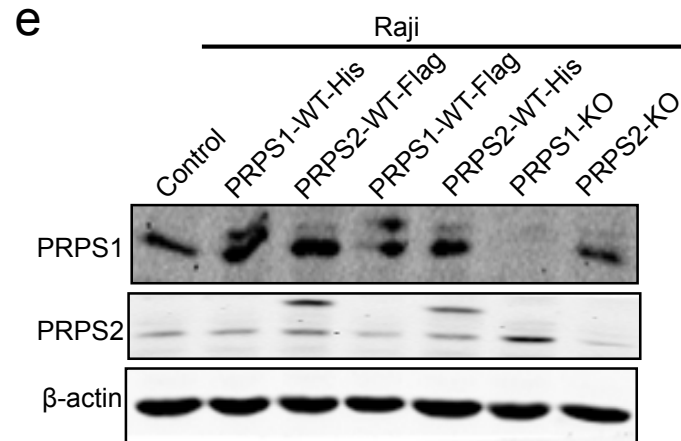

f

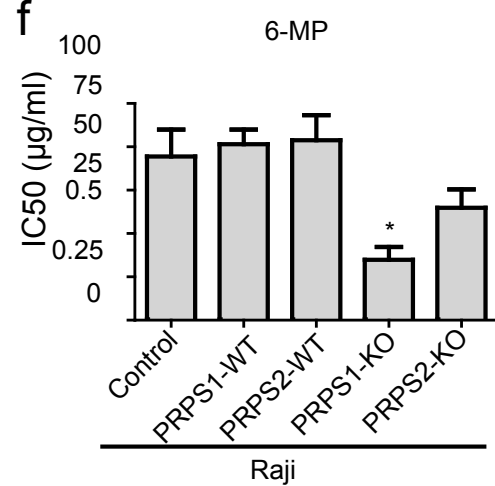

Supplement: Supplementary file 3 — Fig S3 [file JCMM-24-6704-s003.pdf]

Fig.S4

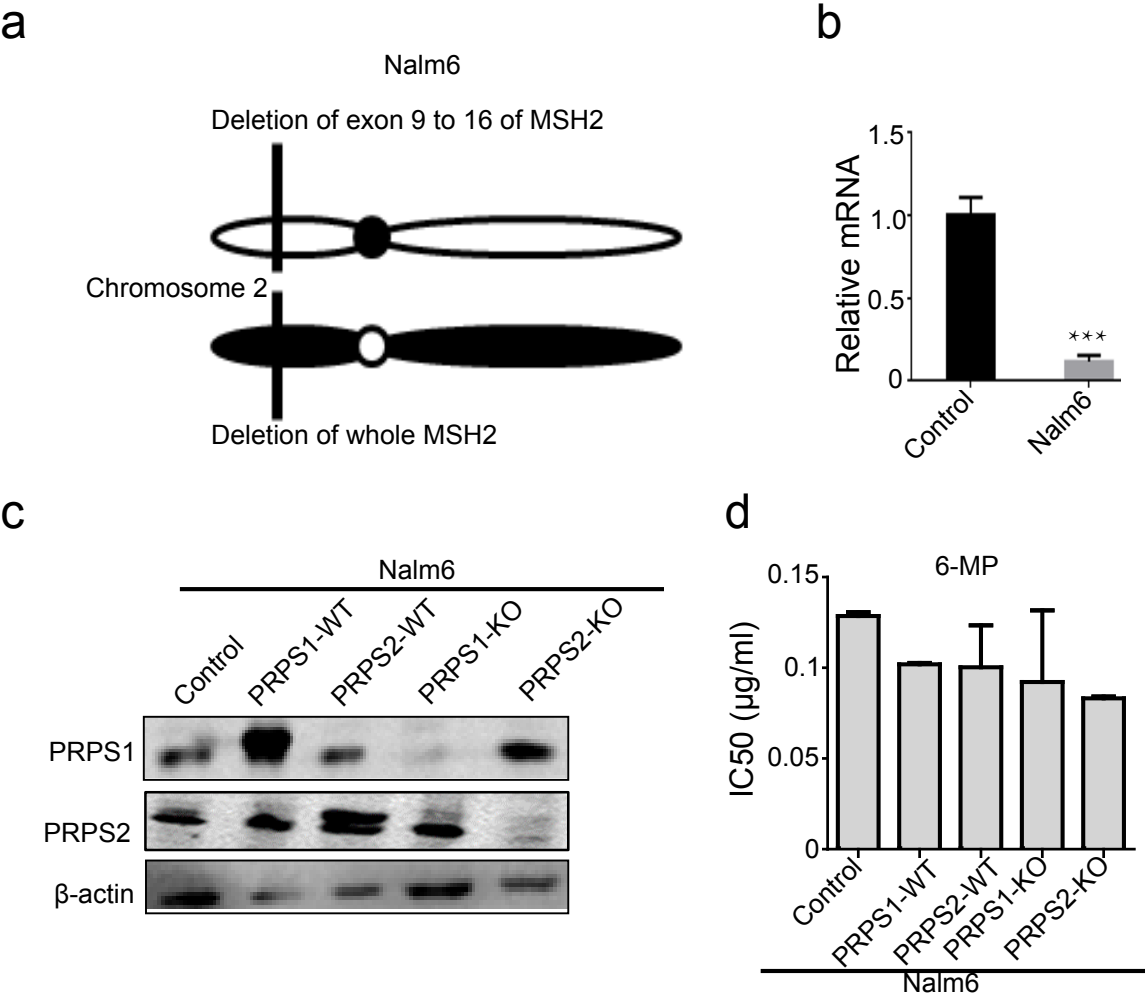

Supplement: Supplementary file 4 — Fig S4 [file JCMM-24-6704-s004.pdf]
